# Inactivation of class II transactivator by DNA methylation and histone deacetylation associated with absence of HLA-DR induction by interferon-*γ* in haematopoietic tumour cells

**DOI:** 10.1038/sj.bjc.6601602

**Published:** 2004-02-17

**Authors:** Y Morimoto, M Toyota, A Satoh, M Murai, H Mita, H Suzuki, Y Takamura, H Ikeda, T Ishida, N Sato, T Tokino, K Imai

**Affiliations:** 1 First Department of Internal Medicine, Sapporo Medical University, South 1, West 17, Chuo-ku, Sapporo 060-8543, Japan; 2 Department of Molecular Biology, Cancer Research Institute, Sapporo Medical University, South 1, West 17, Chuo-ku, Sapporo 060-8543, Japan; 3 Department of Pathology, Sapporo Medical University, South 1,West 17, Chuo-ku, Sapporo 060-8543, Japan

**Keywords:** transcriptional coactivator, DNA methylation, histone acetylation

## Abstract

By presenting immunogenic peptides at the cell surface, major histocompatibility complex (MHC) class II molecules play a key role in the control of adaptive immune responses. Whether expressed constitutively or induced by interferon-*γ*, expression of MHC class II molecules is regulated via coactivator class II transactivator (CIITA); moreover, suppression of their expression is one mechanism by which cancer cells escape host immunity. In this study, we surveyed the relationship between the expression of one MHC class II antigen, HLA–DR, and its coactivators in a group of haematopoietic cell lines, and explored the role of the aberrant DNA methylation in silencing HLA-DR expression. Among 26 cell lines studied, HLA-DR expression was lost from eight T-cell and two myeloid leukaemia cell lines, and this loss was closely associated with suppression of CIITA-PIV expression. Notably, nine of the 10 cell lines that lost CIITA-PIV expression showed methylation of the gene's 5′ CpG island. Thus, DNA methylation is believed to inhibit the expression of MHC class II molecules in haematopoietic tumour cells by silencing its coactivator, CIITA-PIV. Furthermore, methylation of CIITA-PIV was detected in seven of 32 primary acute myeloid leukaemia specimens, indicating that epigenetic alteration is not a cell line-specific phenomenon. Collectively, these data suggest that, by suppressing expression of MHC class II molecules, epigenetic inactivation of CIITA provides a survival advantage to a subset of haematopoietic tumours.

By presenting immunogenic peptides at the cell surface, major histocompatibility complex (MHC) class II molecules play a key role in the control of adaptive immune responses (Cresswell, 1994). Moreover, increasing evidence indicates that the ability of CD4+ T-cells to recognise tumour cells by the MHC class II molecules on their surface enables CD4+ T-cells to contribute to antitumour immune responses; consequently, the absence of MHC class II molecules likely diminishes the antitumour activity of CD4+ T-cells (Ossendorp *et al*, 2000; Wang, 2001). Constitutive expression of MHC class II is limited to specific immune cell types, such as antigen-presenting and dendritic cells. However, their expression can be induced in nonimmune cells that do not constitutively express them by interferon-*γ* (IFN-*γ*).

Expression of MHC class II molecules is also regulated by class II transactivator (CIITA) (Mach, 1999), which does not bind to DNA but acts as a coactivator, interacting with DNA-binding transcription factors such as regulatory factor X (RFX) family, NF-Y and CREB (Scholl *et al*, 1997; Zhu *et al*, 2000). Transcription of CIITA is regulated by CIITA promoters (CIITA-P) I–IV, each of which directs the expression of a unique first exon (Muhlethaler-Mottet *et al*, 1997): CIITA-PI is active primarily in dendritic cells and murine macrophages (Waldburger *et al*, 2001); CIITA-PII has been identified only in human cells, and its function remains unknown; CIITA-PIII is primarily responsible for directing constitutive expression of CIITA in B cells, and can drive CIITA expression after IFN-*γ* stimulation in endothelial cells, fibroblasts or melanocytes (Piskurich *et al*, 1998; Deffrennes *et al*, 2001), and CIITA-PIV is a major regulator of IFN-*γ*-inducible expression of CIITA (Muhlethaler-Mottet *et al*, 1997).

Haematopoietic tumours often show genetic alterations such as nonrandom chromosomal translocations and gene deletion or amplification. In addition, epigenetic modifications of the DNA, which do not alter the sequence code, are now recognised to contribute to the malignant phenotype. For instance, somatic changes in the methylation of CpG dinucleotides commonly occur during the pathogenesis of human tumours (Jones and Baylin, 2002). Among haematopoietic tumours, aberrant methylation of promoter-associated CpG islands (CGIs) in oestrogen receptor, p16INK4A, p15INK4B, p73, p57KIP2 and DAP kinase has been observed in acute lymphocytic leukaemia (ALL), acute myeloid leukaemia (AML), malignant lymphoma and multiple myeloma (Herman *et al*, 1997; Katzenellenbogen *et al*, 1999; Garcia-Manero *et al*, 2002a, 2002b; Li *et al*, 2002). Methylation-mediated silencing of genes involved in immune system function has not been reported in haematopoietic tumours, however.

It is now known that some haematopoietic tumour cells do not express the MHC class II antigen HLA-DR (Kraiba *et al*, 1989; Wetzler *et al*, 2003), though the reason why remains unclear. In the present study, we examined expression HLA-DR and coactivators of MHC class II molecules, as well as the effects DNA methylation and histone deacetylation, in a group of human haematopoietic tumour cell lines. Our data suggest that aberrant methylation and histone deacetylation of the region around the transcription start site of CIITA-PIV are closely associated with the absence of IFN-*γ*-induced expression of CIITA-PIV, which in turn causes absence of HLA-DR induction in haematopoietic tumour cells.

## MATERIALS AND METHODS

### Cell lines and specimens

DNA and RNA were prepared from eight T-cell ALL cell lines (Jurkat, Molt4, CCRF-HSB2, CCRF-CEM, SupT1, PEER, TALL1, Molt3), six B-cell ALL cell lines (BALL1, CCRF-SB, TOM1, LB804, NALM21, NAGL-1), five multiple myeloma cell lines (HS-Sultan, KMS-12PE, RPMI8226, KHM-1B, U266B1), four myeloma cell lines transformed by Epstein–Barr virus (TAPC, IM9, KR12, RPMI1788), one malignant lymphoma cell line (Raji) and two myeloid leukaemia cell lines (K562, KG1). TOM1, LB804 and NALM21 were kindly provided by Dr Ken Kondo of Hokkaido University, Dr Pierre G Coulie of Ludwig Institute for Cancer Research and Dr Yoshinobu Matsuo of Hayashibara Biochemical Labs, respectively. Other cell lines were obtained from the American Type Tissue Culture Collection or the Japanese Collection of Research Bioresources (Tokyo, Japan). In addition, 32 primary acute myeloid leukaemia specimens used for methylation analysis were described previously (Toyota *et al*, 2001). To promote the expression of MHC class II molecules, cells were treated with 100 U ml^−1^ of IFN-*γ* for 48 h prior to their harvest. DNA was extracted using the phenol/chloroform method. Total RNA was extracted using Isogen (Nippon Gene, Japan), according to the manufacturer's instructions.

### Bisulphite treatment

For bisulphite-PCR, genomic DNA was initially treated with sodium bisulphite (Sigma), as described previously (Clark *et al*,. 1994), after which 2-*μ*g samples were denatured for 10 min in 2 M NaOH at 37°C before the addition of 30 *μ*1 of 10 mM hydroquinone (Sigma Chemical Co. St Louis, MO, USA) and 520 *μ*l of 3 M sodium bisulphite (pH 5.0). The mixture was then incubated for 16 h at 50°C. The resultant modified DNA was purified using a Wizard DNA Purification System (Promega, Madison, WI, USA), after which it was again treated with NaOH and precipitated. Finally, the DNA precipitate was resuspended in 20 *μ*l of water and stored at –20°C until used.

### Combined bisulphite restriction analysis (COBRA) and bisulphite sequencing

To examine the methylation status of CIITA, COBRA, a semiquantitative analysis of methylation, was carried out as described previously (Xiong and Laird, 1997). Primers for COBRA were designed based on the nucleotide sequences obtained from Genbank (AL591852). Primer sequences are as follows: CIITAGM1-F, 5′-GTAGTTGGGATGTTATTTTTGATAAAG-3′; CIITAGM1-R, 5′-TCTCCCTCCCRCCAACTCT-3′; CIITAGM2-F, 5′-GGTTATATAGTAAGTTTGGGAGGATG-3′; CIITAGM2-R, 5′-CRACCCCRAAACTCTAAACAC-3′. The PCR products were digested with restriction enzymes that cleave CpG sites retained because of methylation. After ethanol precipitation, the DNA was subjected to 3% agarose gel electrophoresis and stained with ethidium bromide. For bisulphite sequencing, the PCR products were amplified using primers CIITA-GM1F and CIITA-SEQR, 5′-ACAATCTCRAAACCTCRATTCTC-3′, and cloned into pCR4 vector using a TOPO-TA cloning Kit (Invitrogen), after which the plasmid DNA was purified using a PI system (Kurabo, Tokyo, Japan). Sequencing was carried out using a BigDye Terminator Kit and an ABI 3100 DNA sequencer (Applied Biosystems).

### RT–PCR

The RNA (5 *μ*g) was reverse-transcribed using Superscript II (Invitrogen) to prepare first-strand cDNA. Expression of CIITA-PI, PIII and PIV and HLA-DR was assessed using the following primers: CIITAPI-F, 5’-ACTTCCAGGCCATCCTGACT-3’; CIITAPI-R, 5’-GTAGAGGCACAGGGGGTCAGC-3’; CIITAPIII-F, 5’-TTCCTACACAATGCGTTGCC-3’; CIITAPIII-R, 5’-TGCTGAACTGGTCGCAGTTGATGG-3’; CIITAPIV-F, 5’- AGACTTGCCGCGGCCCCAGAG-3’; CIITAPIV-R, 5’-GTAGAGGCACAGGGGGTCAGC-3’; HLADR-F, 5’-GCCAACCTGGAAATCATGACA-3’; HLADR-R, 5’-AGGGCTGTTCGTGAGCACA-3’; RFX5-F, 5’-AACCACCTGGAAGAGCACACTGAC-3’; RFX5-R, 5’-CCAGGCAGGGGTGGCATAGA-3’; RFXAP-F, 5’–GTGCAAGAAACACCGCAACAAGAT-3’; RFXAP-R, 5’–CTGCTGTTGTCTTTGCTCCAAAACT-3’; RFXB-F, 5’–CTCCCTGAAGCACTCCACCACTC-3’; RFXB-R, 5’–AGCAGGAAGCGAACGGTCTCAA-3’; RFXANK-F, 5’–AGACCTCATCCAGACCCAGCAGAC-3’ and RFXANK-R, 5’–CTCGCTGCCGGTTGGTGAGA-3’. Controls consisted of RNA treated identically, but without the addition of reverse transcriptase (RT−). The integrity of the cDNA was confirmed by amplifying GAPDH as described previously (Suzuki *et al*, 2002). Samples (10 *μ*l) of the amplified products were then subjected to 2.5% agarose gel electrophoresis and stained with ethidium bromide.

### Flow cytometry

Cells were incubated for 60 min at 4°C, first with anti-HLA-DR monoclonal antibody L243 (ATCC) and then with affinity-purified fluorescein-conjugated goat anti-mouse IgG+IgM (Kirkegaard and Perry Laboratories, Gaithersburg, MD, USA), after which they were analysed in a FACScan flow cytometer (BD Biosciences). At least 30 000 viable cells were studied in each condition. The data were analysed using CELLQUEST software (BD Biosciences).

### Chromatin immunoprecipitation analysis (ChIP)

Cells (1 × 10^6^) were plated on 10-cm dishes, incubated for 24 h, and then treated with mock or 100 U ml^−1^ IFN-*γ* for 48 h. The cells were then exposed to formaldehyde, and chromatin was immunoprecipitated for 16 h at 4°C using antiacetylated histone H3 antibodies (Upstate Biotechnologies, Lake Placid, NY, USA). After immunoprecipitation, the DNA was recovered using agarose beads, incubated with Proteinase-K at 45°C for 1 h, purified using the phenol/chloroform method, and precipitated with ethanol. PCR was carried out with approximately 1/100 of the immunoprecipitated DNA using SYBR Green sequence detection reagents (Applied Biosystems) in a solution containing l00 ng of DNA, 25 *μ*l of SYBR Green PCR Master Mix and 2.5 pmol of each primer. The primers used were 5′-ACAAGCTCCCTGCAACTCAG-3′ (CIITAChIP-F) and 5′-CCACCACGTGCTTTATCAGA-3′ (CIITAChIP-R). In addition, the 5′ region of GAPDH was amplified as an internal control. The PCR cycling protocol entailed one cycle at 95°C for 5 min and 40 cycles at 95°C for 30 s and 60°C for 1 min. Fluorescent signals were detected using an ABI 7000 Prism 7000 (Applied Biosystems), and the accumulation of PCR product was measured in real time as the increase in SYBR green fluorescence. Data were analysed using ABI Prism 7000 SDS Software (Applied Biosystems). Standard curves relating the initial template copy number to the fluorescence and amplification cycle were generated using the amplified PCR product as a template, and were then used to calculate the DNA copy number in each sample. Ratios of the intensities of the CIITA and GAPDH signals served as relative measures of the histone acetylation level of CIITA in each cell line.

## RESULTS

### Expression of HLA-DR in haematopoietic tumour cell lines

**Figure 1:**
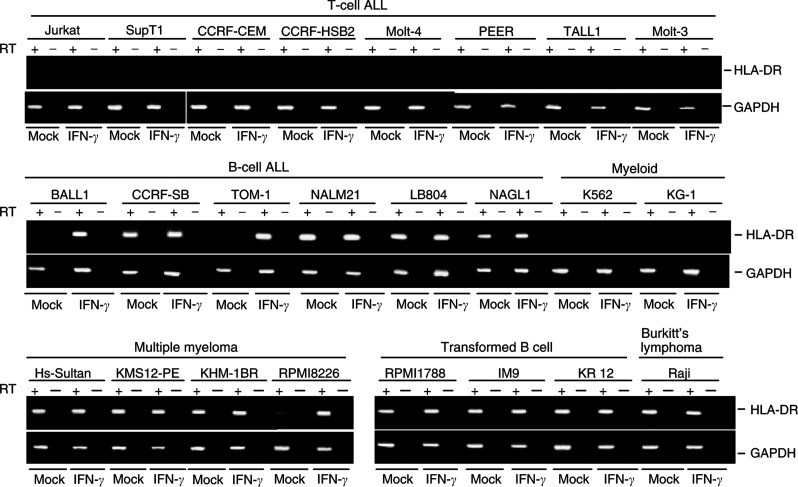
Expression of HLA-DR in haematopoietic tumour cell lines. RT-PCR was carried out using cDNA prepared from haematopoietic tumour cell lines treated with either mock or 100 U ml^−1^ of IFN-*γ* for 48 h. GAPDH was amplified to confirm the integrity of the cDNA. Cell lines and cell types are shown on the top.

We initially examined the expression of HLA-DR in a group of haematopoietic tumour cell lines, with or without IFN-*γ* treatment (Figure 1

, summary in Table 1

**Table 1:** Expression and methylation status of HLA-DR and various types of CIITA in haematopoietic tumour cell lines

	**Expression**								
	**HLA-DR**		**CIITA-PI**		**CIITA-PIII**		**CIITA-PIV**		**Methylation**
**Cell lines**	**Mock**	**IFN-*γ***	**Mock**	**IFN-*γ***	**Mock**	**IFN-*γ***	**Mock**	**IFN-*γ***	**CIITA-PIV**
*B-cell ALL*									
BALL 1	None	+++	None	None	0-+	+	None	+++	Unmethylated
CCRF-S8	+++	+++	None	None	+	+	+++	+++	Unmethylated
TOM 1	None	+++	None	None	None	+	None	+++	Unmethylated
LB804ALL	+++	+++	None	None	+	+	+++	+++	Unmethylated
NALM21 (Ph1+)	+++	+++	None	None	+	+	+++	+++	Unmethylated
NAGL-1	+++	+++	None	None	+	+	+++	+++	Unmethylated
									
*Myeloma*									
KMS12-PE	+++	+++	None	None	None	None	+++	+++	Unmethylated
RPMI8226	+	+++	None	None	None	None	+	+++	Unmethylated
HS-sultan	+++	+++	None	None	None	None	+++	+++	Unmethylated
KHM-1B	+++	+++	None	None	None	None	+++	+++	Unmethylated
U266B1	+++	+++	None	None	None	None	+++	+++	Unmethylated
									
*Mature B cell*									
TAPC	+++	+++	None	None	None	None	+++	+++	Unmethylated
RPMI1788	+++	+++	None	None	+	+	+++	+++	Unmethylated
IM9	+++	+++	None	None	+	+	+++	+++	Unmethylated
KR12	+++	+++	None	None	+	+	+++	+++	Unmethylated
									
T-cell ALL									
Jurkat	None	None	None	None	None	None	None	None	Methylated
Motl4	None	None	None	None	None	None	None	0-+	Methylated
CCRF-HSB2	None	None	None	None	None	None	None	0-+	Methylated
CCRF-CEM	None	None	None	None	None	None	None	0-+	Methylated
SupT1	None	None	None	None	None	None	None	None	Methylated
PEER	None	None	None	None	None	None	None	0-+	Methylated
TALL1	None	None	None	None	None	None	None	None	Methylated
Molt3	None	None	None	None	None	None	None	None	Unmethylated
									
*Burkitt's*									
Raji	+++	+++	None	None	+	+	+++	+++	Unmethylated
									
*Myeloid*									
K562	None	None	None	None	None	None	None	+	Methylated
KG-1	None	None	None	None	None	None	None	None	Methylated

+++: strong expression. +: weak expression. None: no expression.

). RT–PCR analysis of 26 cell lines showed that 13 expressed HLA-DR constitutively; three expressed it following induction with interferon-*γ*; and 10 did not express it at all. None of the T-cell leukaemia cell lines expressed HLA-DR, while cell lines established from B-cell ALL, mature B-cells and Burkit lymphoma all showed constitutive or IFN-*γ*-inducible HLA-DR expression.

**Figure 2:**
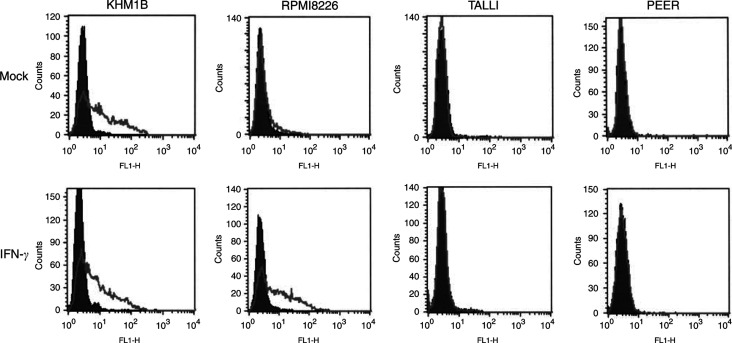
Cell surface expression of the HLA-DR protein in haematopoietic tumour cell lines. Cell lines were treated with either mock or 100 U ml^−1^ IFN-*γ* for 48 h, after which HLA-DR expression was analysed by flow cytometry.

Flow-cytometric analysis showed HLA-DR protein to be present at the surface of cells that express HLA-DR mRNA constitutively (Figure 2

, KHM1B) or after induction with IFN-*γ* (RPMI8226). Conversely, surface expression of HLA-DR was not detected in cells that did not express HLA-DR mRNA (TALL1 and PEER), which indicates that the absence of surface HLA-DR protein was caused by loss of gene transcription, rather than by post-transcriptional alteration.

### Expression of the transactivator of MHC class II in haematopoietic tumour cells

**Figure 3:**
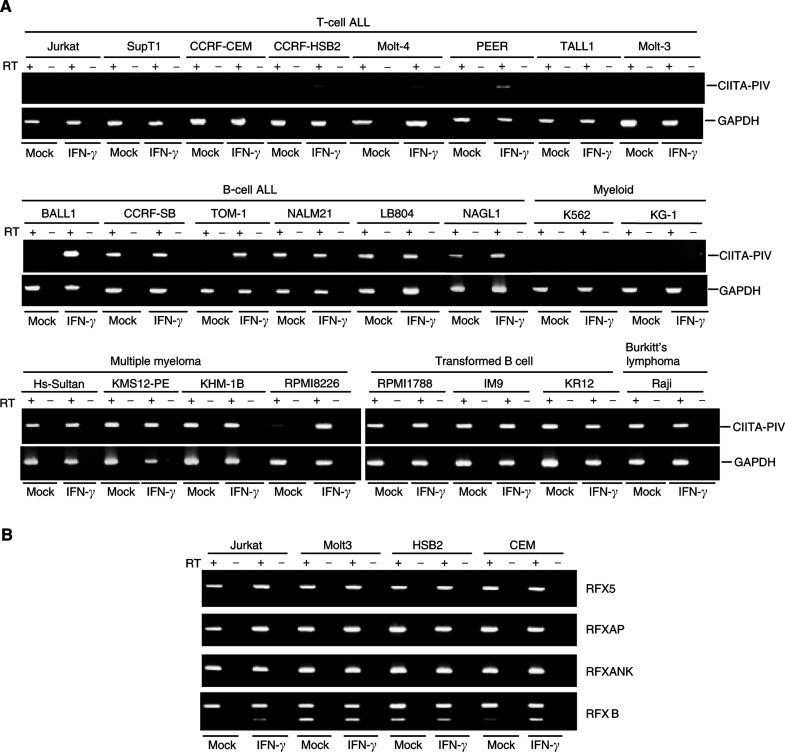
Expression of MHC class II transactivator in haematopoietic tumour cell lines. (**A**) RT–PCR analysis of CIITA expression in haematopoietic tumour cell lines. cDNA from cells treated with either mock or IFN-*γ* for 48 h was amplified using primers that specifically amplify CIITA-PIV. The cell lines analysed are shown on the top. (**B**) RT–PCR analysis of RFX-5, RFX-AP, RFX-ANK and RFX-B in haematopoietic tumour cell lines.

Bare lymphocyte syndrome (BLS) is a severe immunodeficiency caused by the absence of MHC class II molecules (Klein *et al*, 1993). Patients with this syndrome carry a defect in the genes encoding the components of the RFX complex and CIITA (Steimle *et al*, 1993; Villard *et al*, 1997; Masternak *et al*, 1998; Nagarajan *et al*, 1999). To determine the role of RFX and CIITA genes in the suppression of HLA-DR expression, we carried out RT–PCR with a group of haematopoietic tumour cell lines that either did or did not express HLA-DR (Figure 3A and B

). We found ubiquitous expression of all the four RFX subunits in T-cell lines, irrespective of whether they expressed HLA-DR, and therefore excluded RFX inactivation as a cause of the MHC class II suppression (Figure 3B).

When we then examined the expressions of CIITA-PI, CIITA-PIII and CIITA-PIV in each cell line (Figure 3A and Table 1), we found that, as expected, CIITA-PI mRNA was not expressed in any of the haematopoietic cells studied. T-cell ALL and myeloid leukaemia cell lines showed complete suppression of both CIITA-PI and CIITA-PIII, five cell lines (Molt-4, CCRF-HSB2, CCRF-CEM, PEER and K562) expressed low or negligible levels of CIITA-PIV, and five (Jurkat, SupT1, TALL1, Molt-3, and KG1) did not express it at all. Class II transactivator-PII was not examined, as its function remains unknown.

Most of the cell lines derived from multiple myeloma showed constitutive expression of HLA-DR, which correlated with CIITA-PIV expression. Class II transactivator-PIII was expressed in all transformed B-cells, but not in multiple myeloma cells (data not shown). One cell line, RPMI 8226, showed enhanced expression of HLA-DR and CIITA-PIV. This is thought to reflect both the IFN-*γ* inducibility and CIITA-PIV dependence of HLA-DR expression in these cells. Thus, all of the cell lines from B-cell lineages showed strong constitutive and/or inducible HLA-DR expression.

### DNA methylation of CIITA-PIV

**Figure 4:**
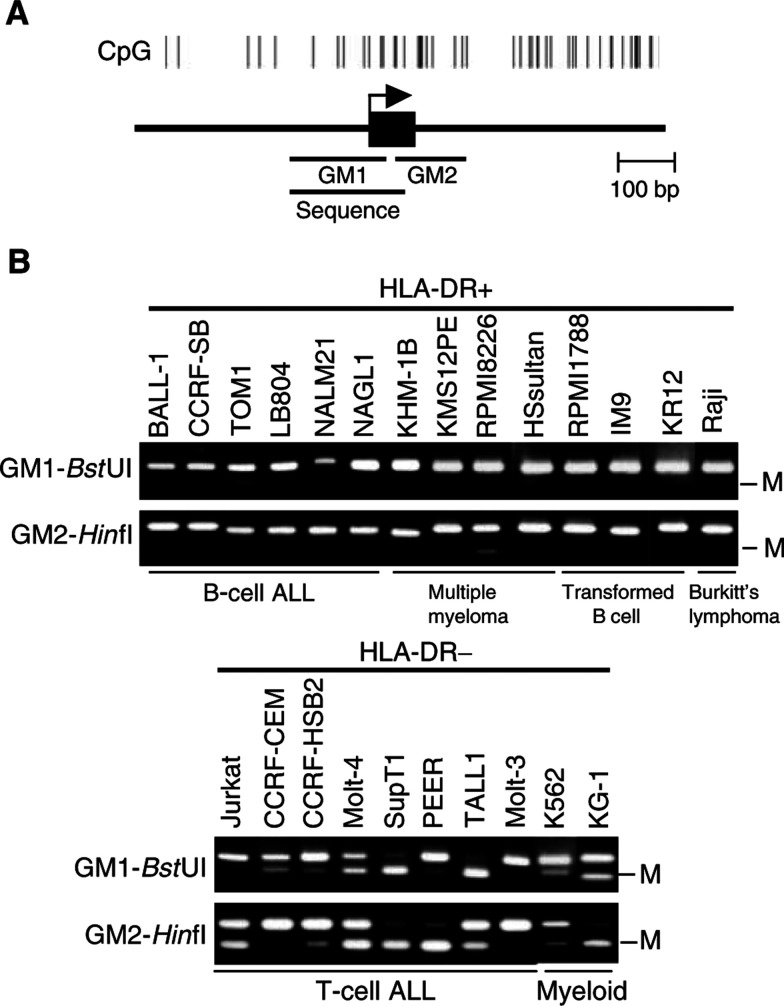
Analysis of CIITA methylation in haematopoietic tumour cell lines by COBRA. (**A**) CpG island of CIITA: exon 1 is indicated by the solid box; the transcription start site is indicated by an arrow; and the region analysed by bisulphite-PCR is indicated by a bar. (**B**) Aberrant methylation of CIITA in haematopoietic tumour cell lines. Cell lines are indicated on the top.

We evaluated the possible involvement of DNA methylation in the silencing of the CIITA-PIV gene, using COBRA (Figure 4

). Cell lines derived from T-cell ALL showed varying degrees of methylation: four (SupT1, Molt-4, PEER and TALL1) showed strong DNA methylation; three (Jurkat, CCRF-HSB2 and CCRF-CEM) showed weak-to-moderate methylation and one (Molt-3) showed no methylation. By contrast, most cell lines of B-cell lineage, including multiple myeloma, show no CIITA-PIV methylation; two (TOM1 and RPMI8226) showed slight methylation. Two myeloid leukaemia cell lines (K562 and KG1) also showed methylation.

**Figure 5:**
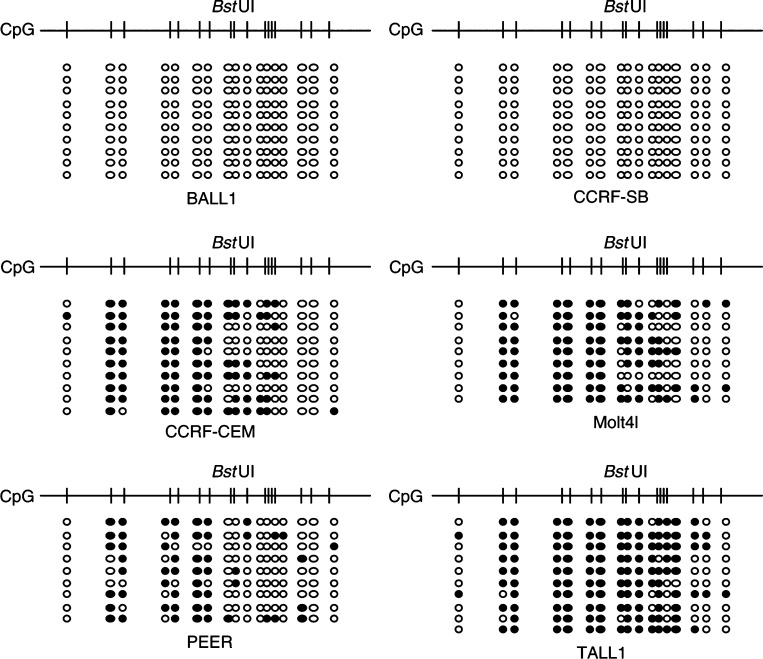
Bisulphite sequencing of CIITA. Amplified PCR products were cloned into pCR4 vector using a TOPO-TA cloning Kit (Invitrogen) and plasmid DNA was purified. Sequencing reaction was performed using a Big-Dye terminator Kit (Applied Biosystems) and electrophoresed using an ABI3100 system (Applied Biosystems). CpG sites are shown above. Methylated alleles are shown as solid circles; unmethylated alleles as shown as open circles.

We then carried out DNA sequence analysis to assess the pattern of methylation in the CIITA-PIV locus using six cell lines, selected because they showed different degrees of methylation (Figure 5

). Cell lines derived from T-cell ALL that do not express CIITA-PIV showed varying levels of DNA methylation in the region analysed. By contrast, no methylation was found in B-cell ALL (BALL1 and CCRF-SB). Careful examination of the pattern of methylation within the loci suggested that upstream regions are more densely methylated than downstream ones.

**Figure 6:**
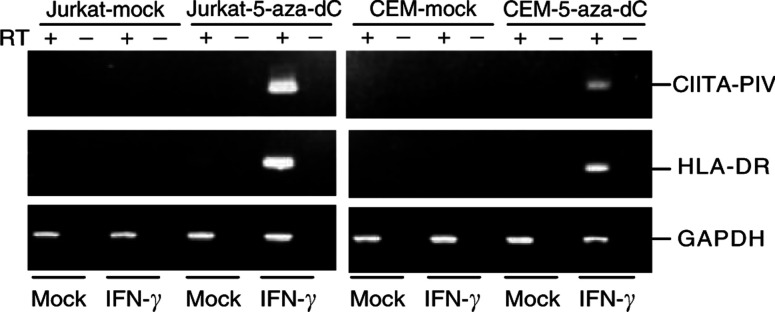
Restoration of IFN-*γ*-induced expression of CIITA and HLA-DR in methylated cell lines. RT–PCR was carried out using cDNA prepared from haematopoietic tumour cell lines treated with either mock, 100 U of IFN-*γ* ml^−1^ of for 48 h, 1 *μ*M 5-aza-dC for 72 h or 1 *μ*M 5-aza-dC and 100 U of IFN-*γ* ml^−1^ for 48 h.

To confirm the role of DNA methylation in gene silencing of CIITA-PIV, two methylated cell lines (Jurkat and CEM) were treated with a methyltransferase inhibitor, 5-aza-dC, and/or IFN-*γ*. Although treating the cell lines with either 5-aza-dC or IFN-*γ* did not restore expression of either CIITA or HLA-DR, combined treatment with both 5aza-dC and IFN-*γ* restored expression of both CIITA-PIV and HLA-DR (Figure 6

).

### Histone deacetylation associated with DNA methylation-dependent CIITA gene silencing

**Figure 7:**
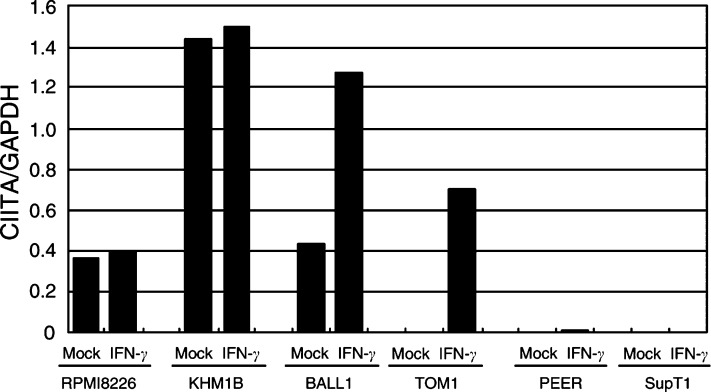
Real-time quantitative PCR analysis of acetylation of the CIITA locus in haematopoietic tumour cell lines. Chromatin immunoprecipitation analysis assays were carried out using antiacetylated histone H3 antibody after treatment with interferon-*γ* or mock. Four cell lines that express CIITA (RPMI8226, KHM1B, BALL1 and TOM1) and two cell lines that do not express (PEER and SupT1) were examined. Fluorescent signals were quantitated using an ABI7000 analysis system (Applied Biosystems). Bars depict the ratios of the intensities of the CIITA and GAPDH signals.

Deacetylation of histone is known to be associated with DNA methylation-dependent gene silencing (Nguyen *et al*, 2001; Suzuki *et al*, 2002). To determine the histone acetylation status in the CIITA locus, we carried out ChIP assays using antiacetylated histone H3 antibody (Figure 7

). Overall, there was an inverse correlaion between DNA methylation and histone acetylation in CIITA-PIV. In unmethylated cell lines, levels of histone acetylation were high in the CIITA promoter region. In BALL1 and TOM1 cells, moreover, histone acetylation was induced by treatment with IFN-*γ*, indicating a role for histone acetylation in gene expression. By contrast, levels of histone acetylation remained low among cell lines that showed CIITA methylation, even after treatment with IFN-*γ*.

### Aberrant methylation of CIITA-PIV in primary AMLs

**Figure 8:**
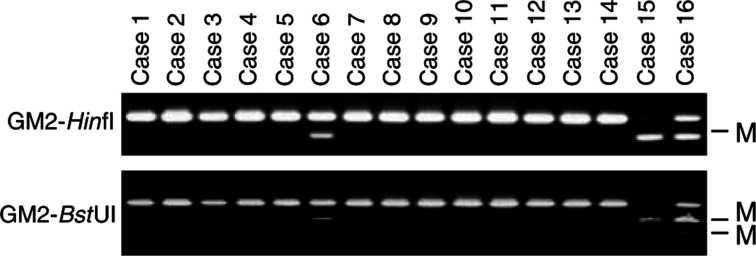
COBRA analysis of CIITA methylation in specimens of AML. Restriction enzymes and the region analysed are shown on the right. Case numbers are indicated on the top.

To determine whether methylation of CIITA-PIV might be a feature of primary leukaemia, we carried out COBRA analyses using DNA from a group of AML specimens. Aberrant methylation of CIITA-PIV was detected in seven of the 32 (22%) cases studied (Figure 8

), whereas no methylation was detected in normal lymphocytes or bone marrow (data not shown). Thus, methylation of CIITA-PIV is not a cell line-specific phenomenon.

## DISCUSSION

To explore the cause of the absence of MHC class II molecules in haematopoietic tumour cells, we examined the relationship between expression of HLA-DR and its co-activators (CIITA and RFX complex). Our results indicate that the CIITA-PIV expression is suppressed by DNA methylation of its 5′ CpG island, which in turn leads to suppression of HLA-DR expression.

DNA methylation is known to be involved in the regulation of CIITA and MHC gene expression in several tumour cell types, including trophoblast-derived cells, a subset of lymphoma cells, and squamous cell carcinoma (Morris *et al*, 2000; Nie *et al*, 2001; Murphy *et al*, 2002; Moreau *et al*, 2003). Our results suggest that DNA methylation of CIITA-PIV is also a major cause of HLA-DR suppression in haematopoietic tumour cells, especially those derived from T-cells and myeloid cells. One exception was Molt-3 T-cell leukaemia cells, which expressed no CIITA-PIV, even though the promoter region of the gene showed no methylation. Further study will be necessary to clarify whether epigenetic mechanisms other than DNA methylation – for example, histone modification (Jenuwein and Allis, 2001) – played a role in the silencing CIITA-PIV expression in those cells. In contrast to T-cells, most of those derived from B-cells showed strong constitutive or inducible HLA-DR expression. One plausible explanation is that constitutive expression of CIITA provides access to transcription factors that protect the CpG island from DNA methylation.

Although expressions of CIITA-PIV and HLA-DR were mutually exclusive, we did not formally exclude the possibility of an association between other CIITA promoters (e.g., CIITA-PIII) and the absence of MHC class II, and, indeed, CIITA-PIII can drive CIITA expression after IFN-*γ* stimulation in a number of cell types, including endothelial cells, fibroblasts and melanocytes. But while the extent to which deregulation of CIITA-PIII is involved in the silencing of the HLA-DR gene awaits further investigation, the fact that the 5′ region of CIITA-PIII does not contain a CpG island makes it unlikely that DNA methylation plays a role in gene silencing.

MHC class II expression has been shown to be inversely related to tumorigenicity and directly related to immunogenicity of mouse L1210 lymphoma clones (Fuji and Iribe, 1986). Indeed, it is now known that the loss of MHC molecules is one of the mechanisms by which cancer cells escape host immunity (Garrido and Algarra, 2001). That most cells of B-cell lineage express HLA-DR, while those of T-cell lineage do not, may be one of the reasons for the difference in prognosis between B-cell and T-cell malignancies – that is, leukaemias or lymphomas involving B-cell lineage generally have better prognoses than their T-cell counterparts. It is therefore noteworthy that epigenetic modifications are reversible, which may make them an effective target for therapeutic intervention. Although intensive chemotherapy remains the therapeutic mainstay for acute leukaemia, long-term cure rates with chemotherapy alone remain at approximately 50%, creating an urgent need for better therapies. Clinical trials are currently underway to study the effects of DNA methylation inhibitors in both haematological malignancies (Lubbert, 2000) and solid tumours (Momparler *et al*, 2000).

Our data suggest that drugs able to reverse aberrant epigenetic modification harbour a therapeutic potential related to their ability to reactivate silenced MHC class II expression. In that regard, expression of HLA-G, an MHC class I molecule, was previously re-established using a demethylating agent or histone deacetylase inhibitors (Moreau *et al*, 2003). Similar results might be expected in certain MHC class II-negative haematologic malignancies (e.g., T-cell ALL), perhaps leading to improved prognoses. Other genes that show aberrant DNA methylation of the promoter-associated CGI in T-cell ALL include p15, p73 and C-ABL (Garcia-Manero *et al*, 2002a, 2002b). Similarly, an association may exist between CIITA-PIV methylation and poorer clinical prognosis. Further evaluation of this association would seem warranted.

The methylation around the transcription start site, which silenced CIITA-PIV gene expression, was in turn inversely related to histone acetylation, indicating a possible role for histone deacetylation in the gene silencing. This would be consistent with the findings of Morris *et al* (2002), who reported histone acetylation to be involved in the transcriptional regulation of CIITA.

Most multiple myeloma cell lines showed strong constitutive expression of HLA-DR. B lymphocytes constitutively express MHC class II genes; however, that expression is downregulated as they differentiate into plasma cells (Halper *et al*, 1978; Dellabona *et al*, 1989), reflecting diminished CIITA gene transcription (Silacci *et al*, 1994). Unexpectedly, myeloma cells, the malignant counterpart of plasma cells, showed constitutive MHC class II expression. Apparently, during the neoplastic change from plasma to myeloma cells, they regain constitutive MHC class II expression. Nonetheless, CIITA-PIII, which normally directs constitutive MHC class II expression in B-cells, remained suppressed in all myeloma cell lines. In this case, constitutive HLA-DR expression was dependent on CIITA-PIV expression. This is of particular interest to us, because the dependence of constitutive MHC class II expression on CIITA-PIV has not been reported previously. As a consequence, multiple myeloma cells showed expression of HLA-DR with no methylation in CIITA-PIV, perhaps making them a good target for CD4+ T-cells, which recognise antigen restricted by MHC class II molecules.

In summary, we have shown that the aberrant methylation of CIITA-PIV is associated with the silencing of CIITA-PIV and HLA-DR expression in T-cell ALL. As this epigenetic change may enable the cells escape from the host immune system, it may be a useful indicator of prognosis. Moreover, drugs that reverse such aberrant methylation would seem potentially useful in the treatment of T-cell ALL.
