# Inactivation of class II transactivator by DNA methylation and histone deacetylation associated with absence of HLA-DR induction by interferon-*γ* in haematopoietic tumour cells

**DOI:** 10.1038/sj.bjc.6602287

**Published:** 2004-11-23

**Authors:** Y Morimoto, M Toyota, H Ikeda, T Tokino, K Imai

**Correction to**: *British Journal of Cancer* (2004) **90**: 844–852. doi:10.1038/sj.bjc.6601602

Owing to an author error, the author affiliations in the above paper were shown incorrectly. The correct affiliations are given below:

Y Morimoto^1^, M Toyota^1,2^, H Ikeda^3^, T Tokino^2^ and K Imai^1^

^1^First Department of Internal Medicine, Cancer Research Institute, Sapporo Medical University, Sapporo 060-8543, Japan; ^2^Department of Molecular Biology, Cancer Research Institute, Sapporo Medical University, Sapporo 060-8543, Japan; ^3^Department of Pathology, Sapporo Medical University, Sapporo 060-8543, Japan

